# The pharmacodynamic and mechanistic foundation for the antineoplastic effects of GFH009, a potent and highly selective CDK9 inhibitor for the treatment of hematologic malignancies

**DOI:** 10.18632/oncotarget.28543

**Published:** 2023-12-20

**Authors:** Fusheng Zhou, Lili Tang, Siyuan Le, Mei Ge, Dragan Cicic, Fubo Xie, Jinmin Ren, Jiong Lan, Qiang Lu

**Affiliations:** ^1^GenFleet Therapeutics (Shanghai) Inc., Shanghai 201203, P.R. China; ^2^Sellas Life Sciences Group, New York, NY 10036, USA

**Keywords:** GFH009, CDK9, cancer, leukemia, cell cycle

## Abstract

To evade cell cycle controls, malignant cells rely upon rapid expression of select proteins to mitigate proapoptotic signals resulting from damage caused by both cancer treatments and unchecked over-proliferation. Cyclin-dependent kinase 9 (CDK9)-dependent signaling induces transcription of downstream oncogenes promoting tumor growth, especially in hyperproliferative ‘oncogene-addicted’ cancers, such as human hematological malignancies (HHMs). GFH009, a potent, highly selective CDK9 small molecule inhibitor, demonstrated antiproliferative activity in assorted HHM-derived cell lines, inducing apoptosis at IC50 values below 0.2 μM in 7/10 lines tested. GFH009 inhibited tumor growth at all doses compared to controls and induced apoptosis in a dose-dependent manner. Twice-weekly injections of GFH009 maleate at 10 mg/kg significantly prolonged the survival of MV-4-11 xenograft-bearing rodents, while their body weight remained stable. There was marked reduction of MCL-1 and c-MYC protein expression post-drug exposure both *in vitro* and *in vivo*. Through rapid ‘on-off’ CDK9 inhibition, GFH009 exerts a proapoptotic effect on HHM preclinical models triggered by dynamic deprivation of crucial cell survival signals. Our results mechanistically establish CDK9 as a targetable vulnerability in assorted HHMs and, along with the previously shown superior class kinome selectivity of GFH009 vs other CDK9 inhibitors, strongly support the rationale for currently ongoing clinical studies with this agent in acute myeloid leukemia and other HHMs.

## INTRODUCTION

Cyclin-dependent kinase type 9 (CDK9) is a global transcription regulator and a crucial component of the super-elongation complex that controls RNA polymerase 2 (RNAP2) phosphorylation and transcriptional elongation. CDK9 is an unusual cell division cycle protein-2 (CDC2)-like kinase in that it does not act directly upon the cell cycle, but rather plays a role in cellular differentiation [1, 2]. CDK9 is also involved in the transcriptional regulation of the myeloid leukemia cell differentiation protein-1 (Mcl-1) and cellular myelocytomatosis (c-Myc) genes, thus promoting intrinsic apoptosis induction.

Dysregulation of the CDK9 pathway has been observed in a variety of hematologic malignancies, including acute myelogenous leukemia (AML), chronic lymphocytic leukemia/small-cell lymphocytic lymphoma (CLL/SLL), diffuse large B-cell lymphoma (DLBLC), and acute lymphoblastic leukemia (ALL), making it an attractive target for cancer therapies [2]. CDK9 exerts global transcriptional control by abating the recruitment of key transcription factors to the initiation complex, as well as controlling expression of super enhancer-regulated genes, that are involved in cell identity. Among these are c-Myc, a downstream proto-oncogene involved in cell cycle control, and Mcl-1, which functions as an apoptotic regulator [3]. In healthy cells, CDK9 is required for MYC protein expression and function. In non-proliferating cells, steady-state MYC acts as a global transcription enhancer, kept under tight regulation by growth factors [4]. However, it has been observed that cancers exhibiting elevated MYC expression also demonstrate a global transcriptional amplification linked to increased MYC binding, positive transcription elongation factor b (P-TEFb) recruitment, and release of RNAP2 from pause. These changes result in the genome-wide activation of transcriptional elongation and subsequently drive expression of tumor-specific oncogenes [5].

CDK9 is a critical component of the P-TEFb complex, responsible for phosphorylation of RNAP2 at its C-terminal domain, which in turn enhances productive elongation of several mRNA transcripts and increases production of the cognate protein products [3, 6–8]. Among these are oncogenic products with short half-lives, including MCL-1, B-cell lymphoma 2 type A1 (BCL2A1), mouse double minute-4 (MDM4), inhibitor of apoptosis-stimulating protein of p53 (iASPP), X-linked inhibitor of apoptosis protein (XIAP), and MYC. Tumor cells that become reliant on (or “addicted” to) these products for survival have a heightened sensitivity to CDK9 inhibition. Intermittent, partial, dynamic-over-time inhibition of CDK9 is sufficient to disrupt the oncogenic protection afforded by these short-lived proteins and is especially effective at disrupting MYC function [3]. This intermittent inhibition strategy can allow for anti-cancer activity while minimizing the unacceptable toxicity that besets CDK9 inhibitors with longer half-lives.

The investigational drug GFH009 is a potent and highly selective small molecule inhibitor that binds CDK9, inhibits its kinase activity, and subsequently blocks RNAP2-mediated transcription maturation. GFH009 is of particular therapeutic interest due to its negligible affinity to other CDK family members, especially proteins belonging to related kinomes and members of the G protein-coupled receptor (GPCR) family. Inhibition with GFH009 hampers expression of cell growth-promoting molecular targets downstream of CDK9, including several cancer-protecting anti-apoptotic proteins [3, 6, 8].

**Figure 1:**
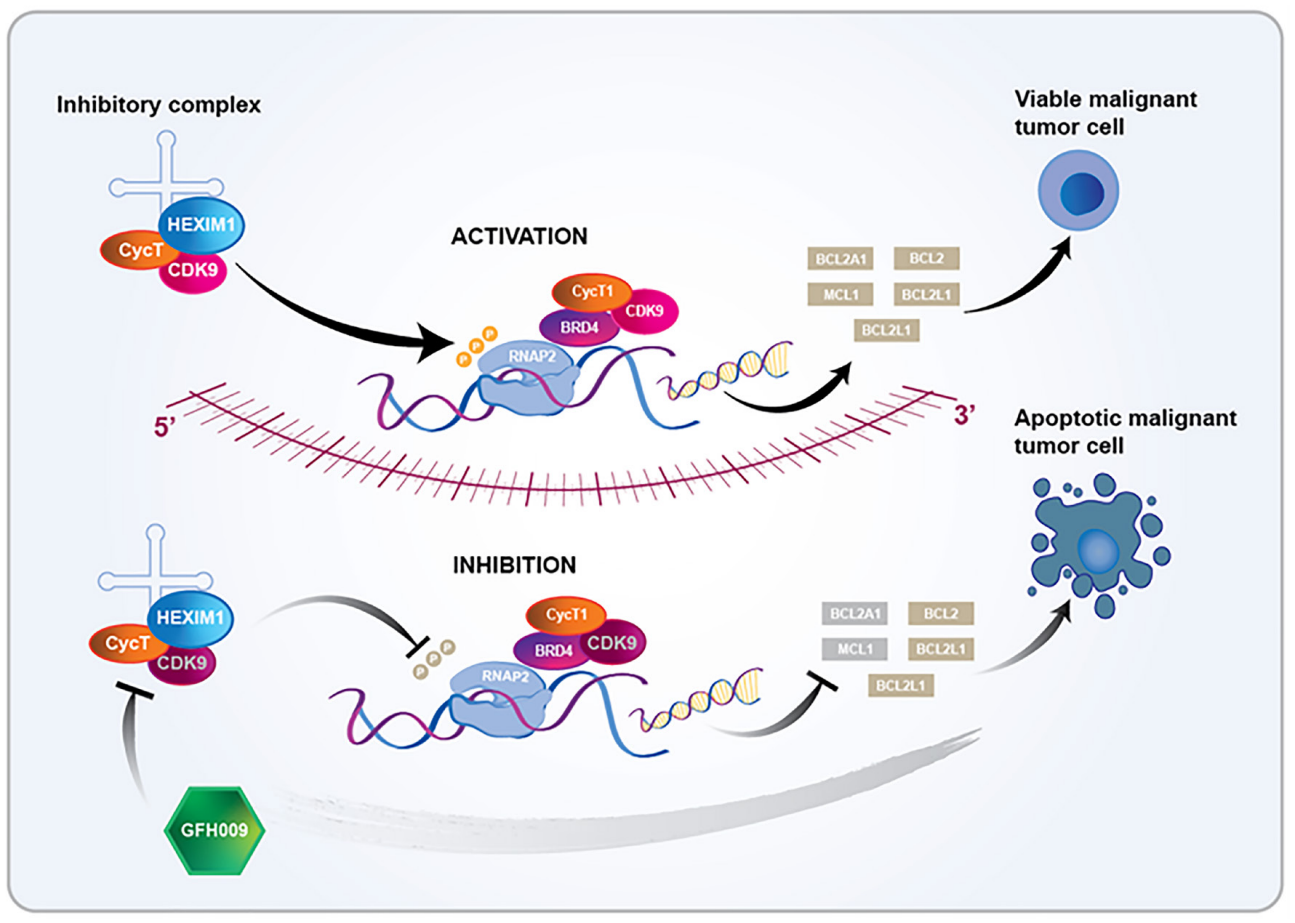
Proposed mechanism of action of the CDK9 inhibitor GFH009 [3, 6, 8].

Inhibition of CDK9 has pleiotropic cellular effects [3, 6, 8]. GFH009 hinders the activity of the inhibitory complex, of which CDK9 is a part, thus repressing the recruitment of key transcription factors to the initiation (super-enhancer) complex. This is accomplished via decreased phosphorylation of the CTD of RNAP2 and results in halting productive elongation of mRNA transcripts. Specifically, the abrupt, dynamic-over-time blocking of the production of short-lived products of oncogenes, such as MCL-1 and BCL2A1, as well as others (MYC, MDM4, iASPP, XIAP) leads to both p53-dependent and -independent, rapidly-induced apoptosis of cells in need of the above group of proteins for their survival and proliferation (Figure 1) [3, 6, 8]. This research aims to summarize current knowledge underlying the mechanism of action (MOA) of GFH009 and explain its robust anti-cancer activity. Understanding GFH009’s MOA allows for a more optimal clinical development path, given the potential for meaningful benefits in patients with hematological malignancies.

## RESULTS

**Figure 2:**
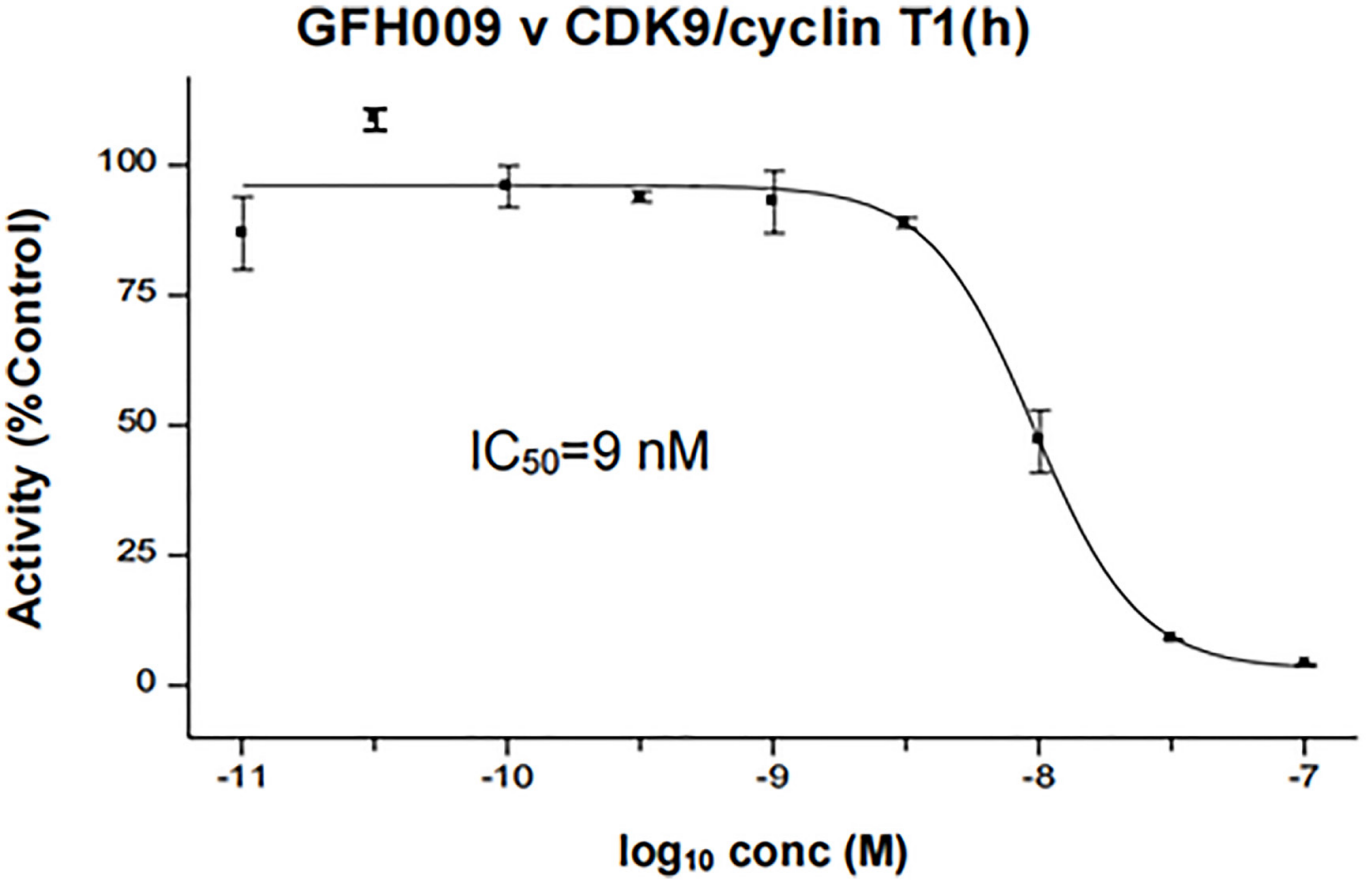
Effect of GFH009 on CDK9 enzymatic activity. IC_50_ of GFH009 inhibition activity against the CDK9/cyclin T1 complex was determined with Eurofin KinaseProfiler radiometric protein kinase activity.

GFH009 is a highly selective inhibitor of CDK9 compared to other CDK family members (see Supplementary Table 1). It does not generally reduce function of GPCR complex members; however, mild inhibition was observed in GPCR panel members M1, M2, M3 and alpha1a at concentrations significantly greater than maximum doses (10 μM; see Supplementary Tables 2, 3). Inhibition of the CDK9/Cyclin T1 complex had a half-maximal inhibitory concentration (IC_50_ of 9 nM) (Figure 2). GFH009 shows negligible effects on other CDK family members (see Supplementary Table 1).

**Figure 3:**
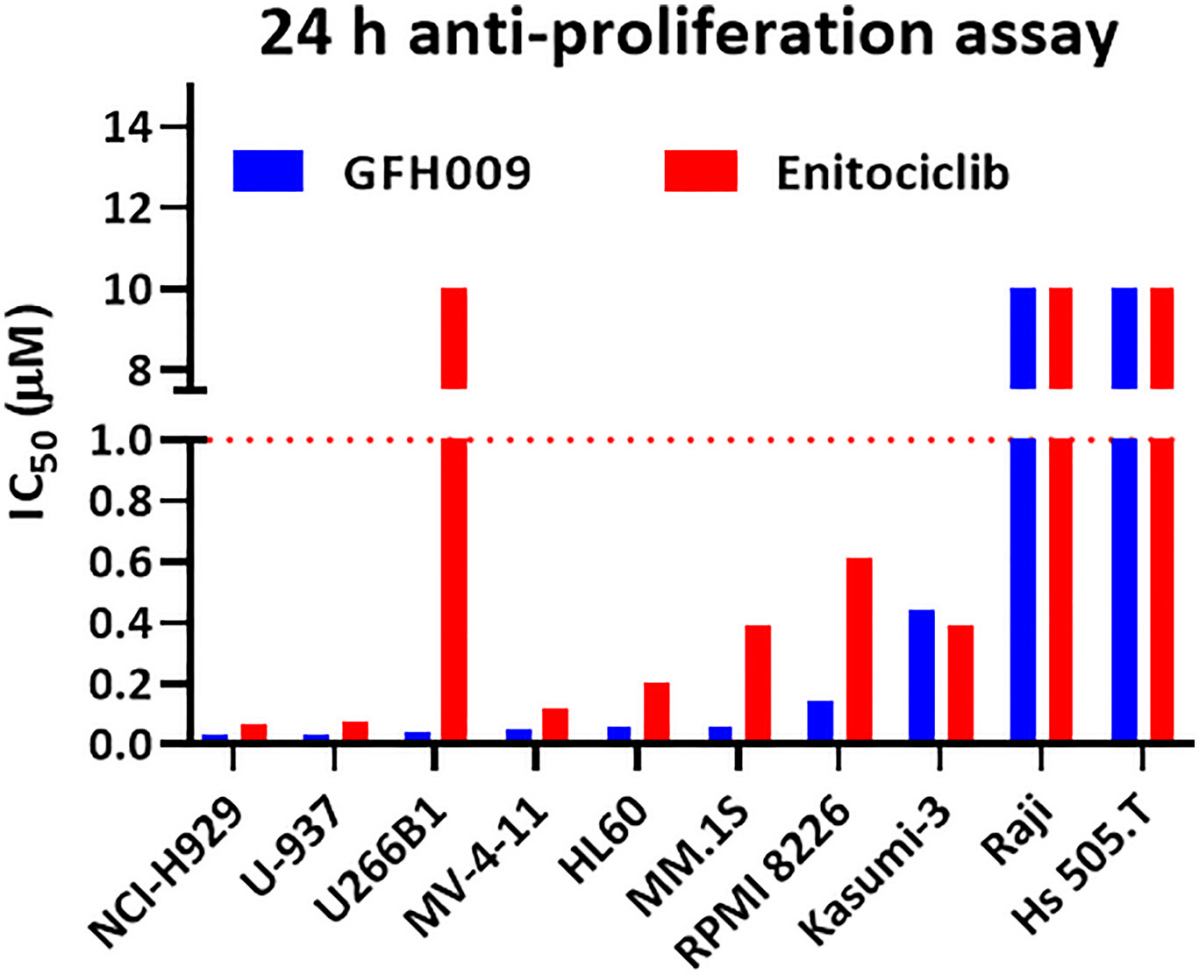
Effect of GFH009 on cell proliferation in assorted human hematologic malignancy cell lines. Cell viability IC_50_s for GFH009 on human hematological malignancy-derived cell lines after 24 hours of exposure to the agent were determined using CellTiter Glo.

GFH009 demonstrated effective antiproliferative activity in a variety of human hematologic malignancy cell lines, with IC_50_ values below 0.2 μM in 7 of the 10 lines tested. Treatment with GFH009 resulted in higher rates of apoptosis than both placebo and the benchmark control compound, enitociclib (Figure 3).

**Figure 4:**
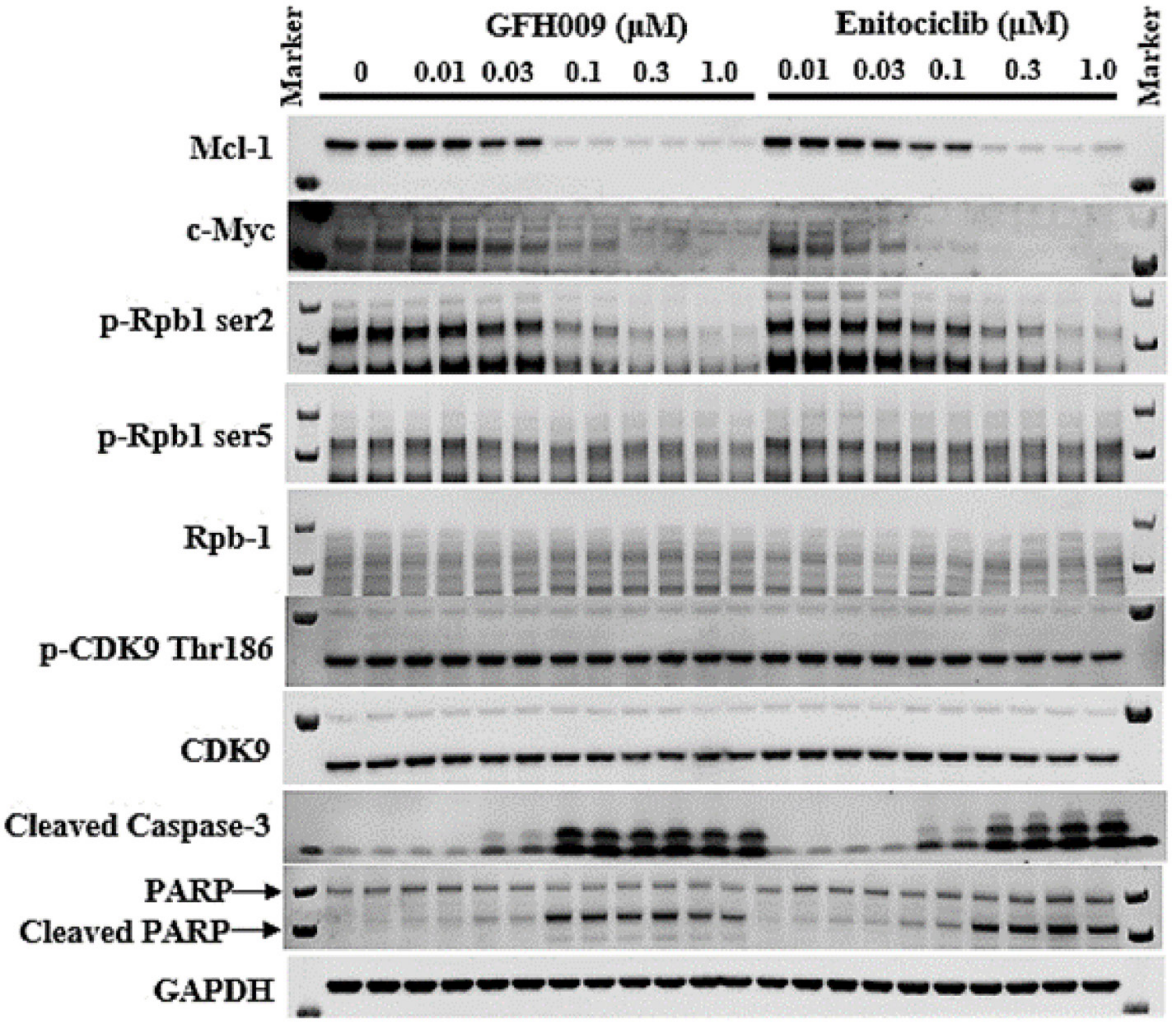
Effect of GFH009 on CDK9-dependent gene expression in cells. Western blot of MV-4-11 cells after exposure to different concentrations of GFH009 or Enitociclib for 4 hours was analyzed. A representative blot was shown from 3 independent experiments.

Western blot analysis of protein expression in MV-4-11 cell cultures following 4-hour incubation with GFH009 demonstrated significant reduction of MCL-1, an anti-apoptotic protein. Expression levels of anti-apoptotic protein MCL-1 and proto-oncogene c-Myc dose-dependently decreased with GFH009 treatment, while the protein expression levels of apoptosis markers Cleaved caspase-3 and Cleaved PARP dose-dependently increased with GFH009 treatment. The expression level of phospho-Rpb1 CTD Ser2, a CDK9 downstream protein related to gene transcription, gradually decreased with increasing concentration of GFH009, while the expression levels of phospho-Rpb1 CTD Ser5 and total Rpb1 were not significantly different from those of the DMSO-only treated control group. GFH009 also induces cleavage of caspase-3 and poly (ADP-ribose) polymerase (PARP) at concentrations greater than 0.1 μM and in a dose-dependent manner at higher concentrations. Both cleaved caspase-3 and PARP are biomarkers for apoptosis and propagate cell death signals through enzymatic activity on downstream molecular targets (Figure 4).

**Figure 5:**
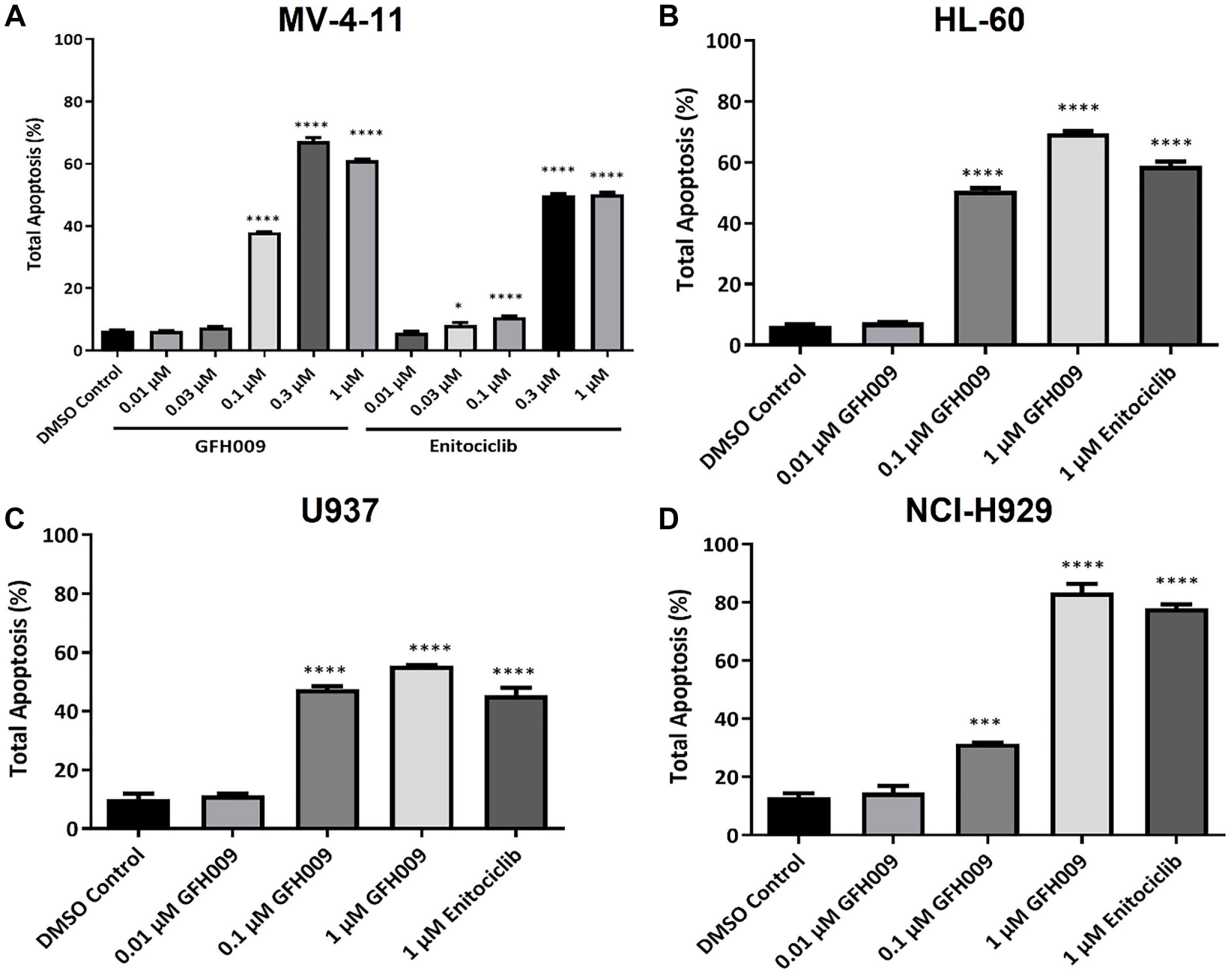
Apoptotic activity of GFH009 in human malignancy cell lines. Four cell lines, MV-4-11 (**A**), HL-60 (**B**), U937 (**C**), and NCI-H929 (**D**) were treated with different concentrations of GFH009 or enitociclib for 6 hours and harvested for cell apoptosis analysis using Annexin V/PI double staining. Data are presented as mean (SD) from multiple independent experiments. ^*^ *P* < 0.05, ^***^ *P* < 0.001, ^****^ *P* < 0.0001, one-way ANOVA with Dunnett’s test analysis compared to DMSO control group.

The apoptotic activity of GFH009 was further assessed through *in vitro* studies in human malignancy cell lines MV-4-11, HL-60, U937, and NCI-H929 at a range of dosages. GFH009 induced significant apoptosis in MV-4-11 following 6-hour treatment and did so in a dose dependent manner (Figure 5A). Apoptosis rate in the DMSO-only treated control group was 6.4 ± 0.1%. Rates for the 0.01 μM, 0.03 μM, 0.1 μM, 0.3 μM, and 1 μM treatment groups were 6.3 ± 0.1%, 7.4 ± 0.3%, 38.1 ± 0.1%, 67.4 ± 1.0%, and 61.2 ± 0.3%, respectively. A similar but less dramatic induction of apoptosis was observed in HL-60, U937, and NCI-H929 cell lines (Figure 5B–5D).

**Figure 6:**
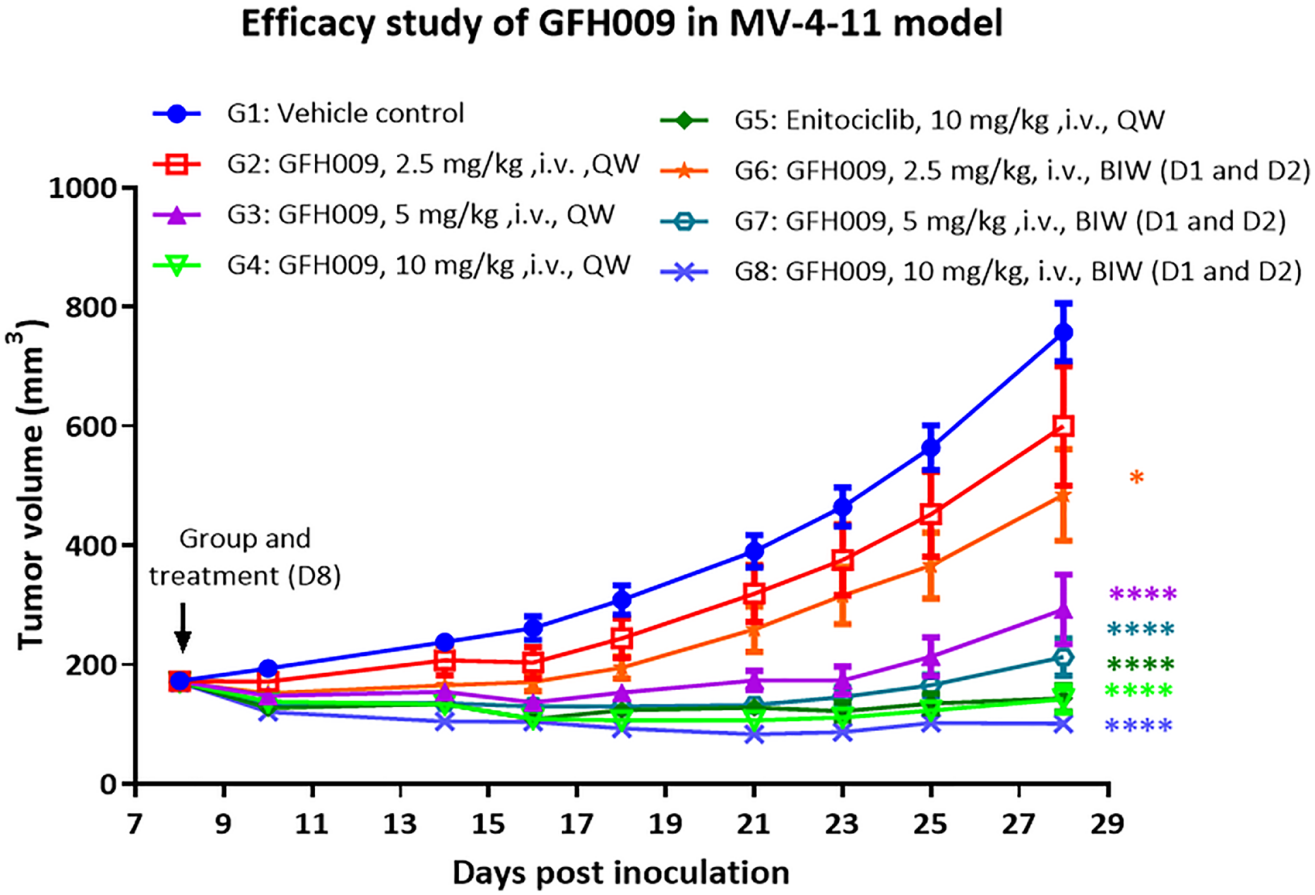
Anti-tumor efficacy of GFH009 in MV-4-11 xenograft model. MV-4-11 xenograft in female BALB/c nude mice were treated with GFH009 or enitociclib at the indicated doses (*n* = 8 mice per group). Results are expressed as mean ± SEM. ^*^ *P* < 0.05, ^****^ *P* < 0.0001, two-way ANOVA with Dunnett’s multiple comparisons test analysis compared to Vehicle control group.

*In vivo* studies in MV-4-11 xenograft mice showed tumor growth inhibition following treatment with GFH009, as measured by total volume, at all doses compared to the vehicle control. Tumor growth inhibition was dose-dependent beginning at 2.5 mg/kg and dosing at 10.0 mg/kg resulted in consistent reduction in total volume through day 29. More frequent dosing (twice weekly vs. once weekly) results in greater tumor growth inhibition at all doses tested (Figure 6). The validation comparator, enitociclib, also inhibited tumor growth.

**Figure 7:**
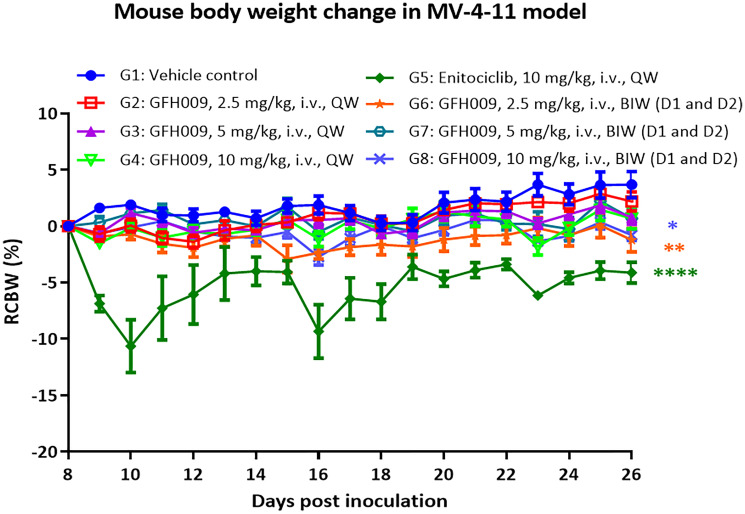
Effect of GFH009 on body weight change in MV-4-11 xenografts. Body weight change of MV-4-11 xenograft (Figure 6) are expressed as mean ± SEM. ^*^ *P* < 0.5, ^**^ *P* < 0.01, ^****^ *P* < 0.0001, two-way ANOVA with Dunnett’s multiple comparisons test analysis compared to the vehicle-treated (control) group.

RCBW% was determined for all animals. During treatment, body mass remained stable in all GFH009 treatment groups, as any relative change was within 5% of the initial body mass measurement through Day 28, post-treatment (Figure 7). During the administration period, the body weight of the mice in the vehicle control group remained stable with a slight increase. Body weight of the mice in the GFH009 maleate treatment groups (whether injected once a week or twice a week) remained stable. In contrast, body weight of the mice in the enitociclib group decreased dramatically after each administration.

**Figure 8:**
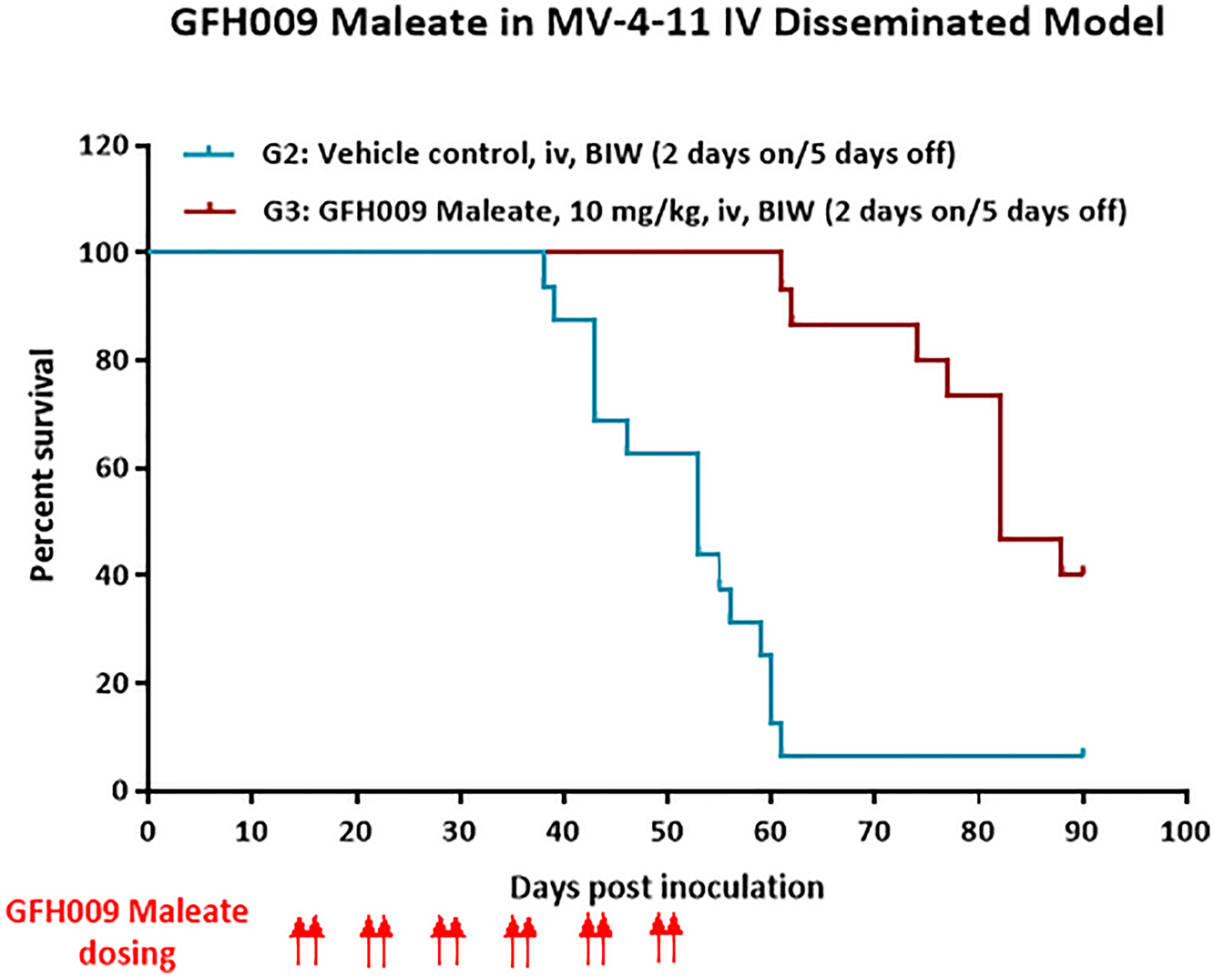
Effect of GFH009 on event free survival of MV-4-11 orthotopic mice following intravenous administration of GFH009 maleate. Female NOD-SCID mice bearing bone marrow-homing MV-4-11 xenografts were treated with either GFH009 Maleate or vehicle at indicated doses for more than 6 consecutive weeks. Animals were monitored through day 90. GFH009 prolonged the survival of MV-4-11-bearing mice compared to vehicle control as shown by the log-rank test (*n* = 16/15 mice per group).

GFH009 maleate, injected twice a week via the tail vein at 10 mg/kg, significantly prolonged the survival of tumor-bearing MV-4-11 mice (*P* < 0.0001) with a hazard ratio of 0.21 and a 95% CI of 0.086 to 0.53. By Day 90, all mice in the cyclophosphamide control group (Group 1, no xenograft cell line injection or GFH009 treatment) survived with an average BW of 22.3 g. Twice-weekly treatment with GFH009 significantly prolonged event-free survival (EFS) in MV-4-11 mice. Mice in the vehicle-treated control group (Group 2), and the GFH009 maleate treatment group (Group 3), showed symptoms such as paralysis and significant BW loss and were subsequently euthanized. At Day 90, only 1 of the 16 mice in the vehicle-treated control group survived (6.3% survival rate). The median survival of the vehicle control group was 53 days. In the GFH009 maleate treatment group, at Day 90, 6 of the 15 mice survived (40% survival rate), with a mean BW of 23.06 ± 0.96 g. Median survival in this group was 82 days (Figure 8). The median event-free survival times (mEFSTs) in both cohorts, as well as the comparison between them by log-rank analysis is shown in Table 1, which denotes a highly statistically significant prolongation of the mEFST of animals treated with GFH009 versus vehicle-treated controls; this due to the beneficial effect of GFH009 in controlling tumor growth.

**Table 1:** Comparison of the median event-free survival time of MV-4-11 orthotopic mice following intravenous administration of GFH009 maleate versus vehicle-treated control animals

Group	EFS (Days)	*P* value (log-rank test)	Hazard ratio (CI 95%)
G2: Vehicle control	53		
G3: GFH009 maleate	82	<0.001	0.22 (0.086, 0.53)

Abbreviations: CI: confidence interval; EFS: event-free survival.

**Figure 9:**
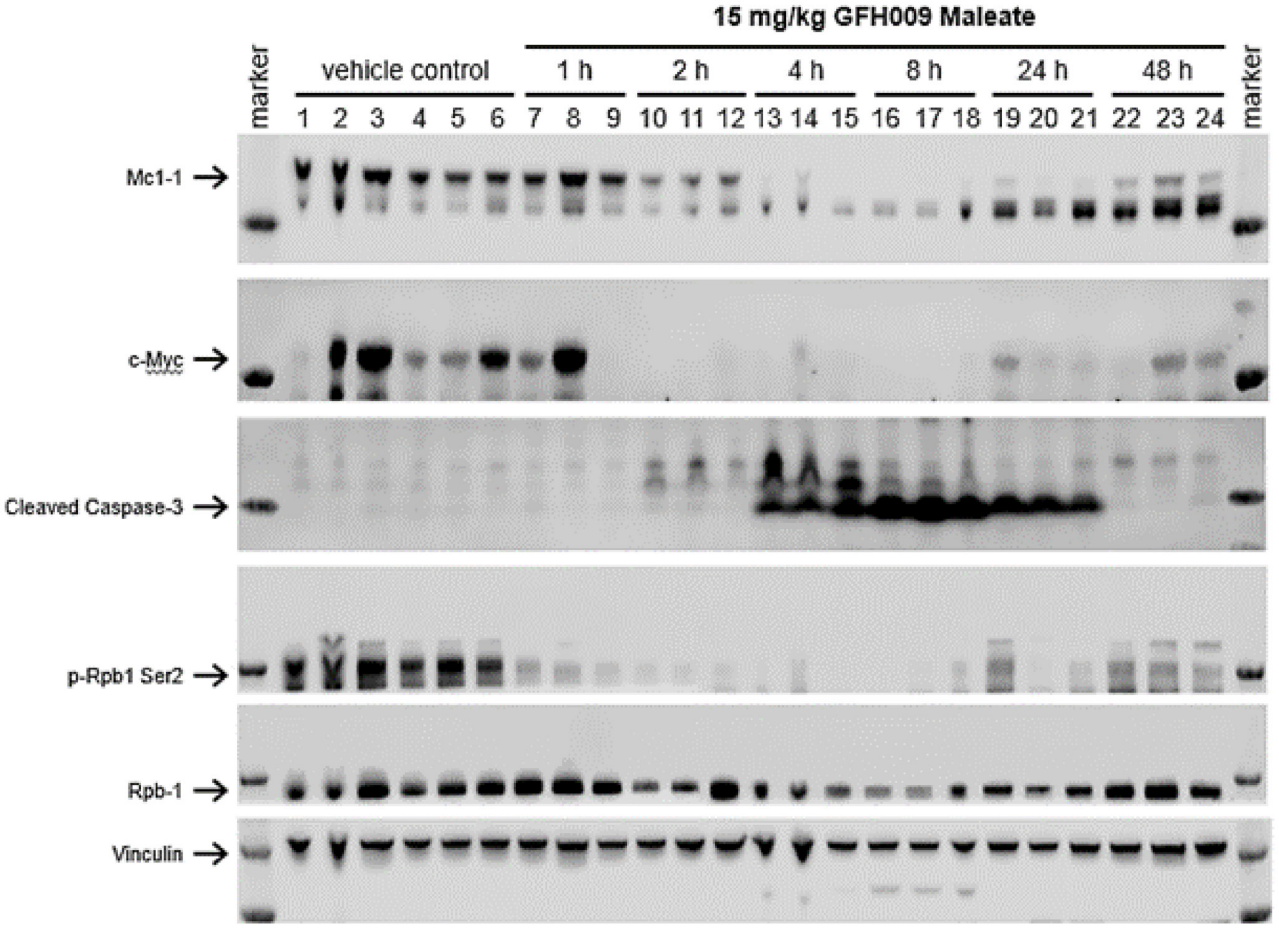
Effect of GFH009 treatment on CDK9-dependent protein expression *in vivo*. Female BALB/c nude mice were subcutaneously inoculated with 5 × 10^6^ MV-4-11 cells. When tumors reached approximately 600 mm^3^ (Day 25), animals were treated with a single dose of vehicle or 15 mg/kg GFH009 Maleate. The mice were euthanized, and tumor tissues were collected at different times after administration as indicated for western blot analysis. Three individual mice were selected from GFH009 treatment group.

In alignment with *in vitro* studies, western blot analysis of protein expression in MV-4-11 xenograft mice indicates that GFH009 reduces the expression of oncogenes MCL-1 and MYC proteins *in vivo* (Figure 9). One hour after a single dose of GFH009, expression of p-Rpb1 phospho-Ser2 began to decrease significantly compared to control and continued through 48 hours post-administration. Levels of the anti-apoptotic protein MCL-1 and oncoprotein MYC began to decrease significantly 2 hours following administration and show continued reduction through the 48-hour time point.

## DISCUSSION

Though several agents targeting CDK9 to inhibit cell growth are being investigated as cancer therapeutics, many show significant cross-reactivity with other members of the CDK family. This non-specificity increases toxicity burden and reduces tolerability, especially in the setting of chronic administration, limiting these compounds’ potential as cancer treatments. GFH009 is a potent and highly selective CDK9 inhibitor that demonstrates phasic (dynamic) – rather than tonic (static) – reduction in the expression of several key downstream targets. Its specific and highly selective inhibitory action disrupts expression of oncogenes that drive signaling pathways crucial for rapid cellular division in malignant cell lines. As tumor stabilization and shrinkage appear to be dose-dependent, it is likely that this downregulation of labile, short-lived oncogene products is the key to GFH009’s ability to inhibit cellular division.

MCL-1 and MYC are proteins that play a crucial role in protecting cells from programmed death. Treatment with GFH009 reduces the expression of both in *in vitro* and *in vivo* studies, indicating that treated cells are likely being ‘forced’ to favor apoptosis. The depletion of CDK9 by GFH009 provides further evidence that this compound inhibits growth by effectively and efficiently depriving oncogene-addicted cancer cells with high rates of DNA transcription of crucial survival signals. Lacking anti-apoptotic protection as a result of CDK9 inhibition, these hyperproliferating cells are more likely to cease division and progress to apoptosis.

Collectively, these data suggest that GFH009 depletes the protective anti-apoptotic proteins produced downstream of CDK9 in both *in vitro* and *in vivo* (murine) models of hematologic malignancies, meriting further investigation as a potential treatment for hematological cancers, more specifically AML, in clinical settings. Recent investigations of the pharmacokinetic profiles of two formulations of GFH009 maleate (at pH4.5 and pH 6.0) have shown comparable activity following single IV administration in Sprague-Dawley rats. Given that the normal human blood pH range is 7.35 to 7.45, the formulation at pH 6.0 is most appropriate for use in clinical research settings [9].

A phase 1, first-in-human open-label, single-arm dose-escalation and dose-expansion study of monotherapy with IV GFH009 maleate (pH 6.0) began enrolling patients in May 2021 (https://clinicaltrials.gov/ ID: NCT04588922). This study is investigating both the safety and tolerability of GFH009 in patients with relapsed or refractory advanced hematologic malignancies, including AML, CLL/SLL and lymphoma. Clinical investigation of GFH009 is ongoing, however preliminary safety and efficacy results in a cohort of 16 patients with assorted lymphomas have been reported at the 64th American Society of Hematology (ASH) Annual Meeting and Exposition in 2022 [10].

Together, the results of this preclinical investigation program suggest that induction of apoptosis is a key component of GFH009’s anti-tumor mechanism of action, positioning this compound favorably as a potentially promising treatment for not only hematologic malignancies but also solid tumors.

## MATERIALS AND METHODS

### *In vitro* analyses

GFH009 inhibition activity against the CDK9/cyclin T1 complex as well as off-target CDK family reactivity were assayed by Eurofins Pharma Discovery Services UK Ltd., (Dundee, UK). Determination of off-target inhibition of other kinases was conducted by DiscoverX LeadHunter Discovery Services (Freemont, CA, USA.) GFH009 reactivity to GPCR family members was assayed by Wuxi AppTech (Shanghai, China).

The antiproliferative activity of GFH009 was assessed in cultured cell lines derived from human hematological malignancies over a period of 24-hour *in vitro* assay readouts. Enitociclib was used as a comparator for experimental validation (provided by MedChem Express, Monmouth, NJ, USA). Cell growth was analyzed by CellTiter Glo in 96-well plates and detected via chemiluminescent signal. The absolute inhibitory concentration of 50% (AbsIC_50_ values of the drug were calculated by fitting the drug inhibition rate curve with the “log (inhibitor) versus response – variable slope (4 parameters)” model by using the Prism GraphPad 6 for Windows v6.01 software.

The effect of GFH009 on CDK9-regulated signaling pathways in human hematological malignancy cell lines was assessed via western blot. Cultured MV-4-11 cells, derived from human blastic AML cells (ATCC), were incubated at 37°C and 5% CO_2_ for 4 hours at final concentrations of 1 μM, 0.3 μM, 0.1 μM, 0.03 μM, and 0.01 μM for both GFH009 and enitociclib. Protein isolates were collected from pelleted cells as previously described, semi-quantified via western blot and analyzed using Image Studio 5.0 (LI-COR Biosciences, Lincoln, NE, USA).

Initiation of apoptosis by GFH009 was assayed in multiple human cell lines, including MV-4-11, HL-60, U937, and NCI-H929. Cells were incubated at 37°C and 5% CO_2_ for 6 hours at final concentrations of 1 μM, 0.3 μM, 0.1 μM, 0.03 μM, and 0.01 μM for both GFH009 and Enitociclib. After drug treatment, cells were collected, and apoptosis determined using Annexin V-FITC and propidium iodide (PI) staining (Becton, Dickinson and Co, Franklin Lakes, NJ, USA). Samples were examined using an Attune NxT Flow Cytometer (Thermo Fisher Scientific, Waltham, MA, USA) within 1 hour of staining and analyzed using FlowJo vX.0.7 software. The apoptotic rate between groups was statistically compared by means of one-way ANOVA (Dunnett’s multiple comparisons test). Differences from the dimethyl sulfoxide (DMSO)-only treated control group was considered statistically significant at a *P* value of < 0.05. All plotting and statistical analysis were conducted using the Prism GraphPad 6 for Windows v6.01.

### *In vivo* analyses

*In vivo* studies of tumor suppression were conducted in female BALB/c nude mice (Beijing Vital River Laboratory Animal Technology Co, Ltd., Changping, Beijing, China) bearing MV-4-11 xenografts (ATCC, Rockville, MD, USA). Each mouse was inoculated subcutaneously into the right dorsal-region with 5.0 × 10^6^ cultured MV-4-11 cells in a total volume of 0.2 ml solution and allowed to develop xenograft tumors. Once tumor size had reached 130–230 mm^2^, mice were divided into groups to receive 1 of 8 different treatment preparations dosed intravenously via the tail vein (Table 2). GFH009 maleate was dissolved in 50 mM PBS to required concentrations and used immediately after preparation. Enitociclib was administered as a 1.0 mg/mL solution in ultrapure water with 5% DMSO. Vehicle control was 50 mM PBS with no dissolved treatment drug.

**Table 2:** Summary of *in vivo* treatments assessing tumor suppression in GFH009 maleate treated MV-4-11 xenograft model mice

Group	Treatment	Dose			
		*N*	mg/kg	Route	Schedule
1	Vehicle Control	8	–	i.v	BIW
2	GFH009 Maleate	8	2.5	i.v	QW
3	GFH009 Maleate	8	5.0	i.v	QW
4	GFH009 Maleate	8	10.0	i.v	QW
5	Enitociclib	8	10.0	i.v	QW
6	GFH009 Maleate	8	2.5	i.v	BIW
7	GFH009 Maleate	8	5.0	i.v	BIW
8	GFH009 Maleate	8	10.0	i.v	BIW

Abbreviations: BIW: 2 times a week; i.v: intravenous; QW: once a week.

Animals dosed BIW were given treatments on 2 consecutive days. Weights were recorded for each mouse every day and tumor volumes assessed 3 times a week. Total treatment administration occurred across 3 consecutive weeks.

Subcutaneous tumor volume (TV) was calculated as TV = (L × W^2^)/2, where L was the length and W was the width of the tumor [11]. Tumor growth was used to calculate the tumor growth inhibition (TGI), and percent TGI was used as a measure of anti-tumor activity for each treatment. TGI was calculated as TGI (%) = (1 – avTi-0/ avCi-0)) × 100, where avTi-0 is the mean TV of the treatment group on a given day minus the mean TV of this treatment group on the day of group assignment, and avCi-0 is the mean TV of the vehicle control group on a given day minus the mean TV of the vehicle control group on the day of group assignment.

Relative change in body weight (RCBW) was determined as RCBW (%) = (Bwi – BW0)/BW0 × 100, where Bwi is body weight of a given mouse on a given day, and BW0 is initial body weight on the day they were assigned to a treatment group.

Tumor volume data was subjected to a base-10 logarithmic transformation and subsequent comparisons of TV across groups was made using two-way ANOVA (Dunnett’s multiple comparisons test). All plotting and statistical analyses were conducted using GraphPad Prism 6.00 (GraphPad Software).

*In vivo* studies of survival following GFH009 administration were conducted in female NOD-SCID mice (Beijing Vital River Laboratory Animal Technology Co., Ltd., Changping, Beijing, China) bearing bone marrow-homing MV-4-11 xenografts. Mice were inoculated with 0.2 mL of prepared MV-4-11 cells to a final dose of 1 × 10^7^ cells via the tail vein, which eventually lodged and resided mostly in the bone marrow of the mice, akin to an orthotopic AML model. Treatment solution consisted of GFH009 maleate at a concentration of 1.0 mg/mL dissolved in 50 mM PBS. All animals were pretreated with cyclophosphamide for myeloablative purposes at 150 mg/kg administered via intraperitoneal injection for 2 consecutive days. Mice were assigned to 3 dosing groups as described in Table 3. Mice in Group 1 were injected with carrier solvent via tail vein twice a week on 2 consecutive days and discontinued for 5 days for observation. Mice in Groups 2 and 3 were selected 2 weeks after cell inoculation to receive either vehicle carrier alone or GFH009 maleate injection. In both cases, animal subjects received biweekly injections on 2 consecutive days followed by 5 days of discontinuation for observation. All mice were weighed daily, and total treatment administration occurred over 6 consecutive weeks.

**Table 3:** Summary of *in vivo* treatments to determine effect of GFH009 maleate on survival in MV-4-11 bone marrow-homing xenograft model mice

Group	Treatment	Dose			
		*N*	mg/kg	Route	Schedule
1	Control (no cell inoculation)	5	–	i.v	BIW
2	Vehicle control (cell inoculated, no treatment)	16	–	i.v	BIW
3	GFH009 Maleate	15	10.0	i.v	BIW

Abbreviations: BIW: 2 times a week; i.v: intravenous.

Animals were monitored through day 90 and either euthanized at experiment completion or when they showed significant body weight (BW) loss, paralysis, or limited mobility. Kaplan-Meier survival curves of the mice in Groups 2 and 3 were plotted, followed by log-rank analysis to calculate median survival time, *p* value, hazard ratio, and 95% confidence interval (95% CI). Differences were considered statistically significant when *P* < 0.05, with an upper limit of 95% CI <1. All plotting and statistical analysis were conducted using Prism GraphPad 6 for Windows, v8.0.

*In vivo* protein expression was assessed via SDS-PAGE and western blotting of samples from female BALB/c nude mice following treatment with GHF009. Following inoculation with 5.0 × 10^6^ MV-4-11 cells into the right dorsal region, mice were observed daily for symptoms and euthanized when they experienced significant BW loss (RCBW <−20%) or exhibited other severe symptoms. When tumors reached approximately 600 mm^3^ (Day 25), animals were randomly divided into two groups. Group 1 received a single dose of vehicle control. Group 2 received a single dose of GFH009 maleate at 15 mg/kg. Both were administered via tail vein injection.

All mice were euthanized, and tumor tissues collected at required times following administration. Group 1 control animals were euthanized at 2 hours post-administration. Group 2 animals were euthanized at 1, 2, 4, 8, 24, and 48 hours after administration. Tumor tissue was harvested, weighed, and flash frozen in liquid nitrogen. Total proteins were extracted and analyzed by western blot to determine expression of phospho-Rpb1 CTD Ser2, RPB1, MYC, MCL-1, Cleaved Caspase-3, and vinculin. Image Studio V5.0 was used to analyze all western blot membrane images.

## CONCLUSION

The target selectivity of the CDK9 inhibitor GFH009 has the potential to reduce the drug’s toxicity and increase tolerability, making it a promising treatment option for hematological and solid tumor malignancies. In both *in vitro* and *in vivo* studies, GFH009 appears to be an effective CDK9 inhibitor, significantly altering short-lived oncogenic protein expression and inducing apoptosis in cancerous cells. Ongoing clinical trials are further exploring both the efficacy and safety of GFH009 for the treatment of hematologic cancers.

Results from this study were partially presented in an abstract and poster format at the 64th ASH Annual Meeting and Exposition, Dec. 10–13, 2022, New Orleans, LA, USA.

## SUPPLEMENTARY MATERIALS



## Abbreviations

AbsIC50: absolute relative 50% inhibitory concentration
ALL: acute lymphoblastic leukemia
AML: acute myelogenous leukemia
ANOVA: analysis of variance
ASH: American Society of Hematology
BCL2A1: B-cell lymphoma 2 type A1
BIW: twice a week
BW: body weight
CDC2: cell division cycle protein 2
CDK9: cyclin dependent kinase 9
CLL: chronic lymphocytic leukemia
DLBLC: diffuse large B-cell lymphoma
DMSO: dimethyl sulfoxide
FIH: first in human
GPCR: G-protein coupled receptor
iASPP: inhibitor of apoptosis-stimulating protein of p53
IC50: 50% inhibitory concentration
IV: intravenous
MDM4: mouse double-minute-4 (protein)
mEFST: median event-free survival time
PARP: poly (ADP-ribose) polymerase
PBS: phosphate-buffered saline
PI: propidium iodide
P-TEFb: positive transcription factor b
QW: once a week
RCBW: relative change in body weight
RCBW%: rate of change in body weight
RNAP2: RNA polymerase 2
SD: standard deviation
SDS-PAGE: sodium dodecyl sulfate-polyacrylamide gel electrophoresis
SLL: small-cell lymphocytic leukemia
TGI: tumor growth inhibition
TV: tumor volume
XIAP: X-linked inhibitor of apoptosis protein
