# Supplementary material for: The pharmacodynamic and mechanistic foundation for the antineoplastic effects of GFH009, a potent and highly selective CDK9 inhibitor for the treatment of hematologic malignancies
Source: Oncotarget. 2023 Dec 20;14:997–1008. doi: 10.18632/oncotarget.28543 (PMC10732257; doi:10.18632/oncotarget.28543)
Supplement: Supplementary file 1 [file oncotarget-14-28543-s001.pdf]

# The pharmacodynamic and mechanistic foundation for the antineoplastic effects of GFH009, a potent and highly selective CDK9 inhibitor for the treatment of hematologic malignancies

## SUPPLEMENTARY MATERIALS

**Supplementary Table 1: Inhibitory action of GFH009 on other CDK family members**

| Various CDK/ cognate cyclin pairs tested | Remaining enzymatic activity (%) after exposure to GFH009 at a concentration of 1 µM/10 µM | Selectivity vs. CDK9 |
|---|---|---|
| Cdc7/cyclinB1 | 75/47 | >100 |
| CDK1/cyclin B | 97/50 | >1000 |
| CDK2/cyclin A | 80/51 | >1000 |
| CDK2/cyclin E | 63/27 | >100 |
| CDK3/cyclin E | 74/39 | >100 |
| CDK4/cyclin D3 | 70/41 | >100 |
| CDK5/p25 | 79/43 | >100 |
| CDK5/p35 | 66/43 | >100 |
| CDK6/cyclin D3 | 85/58 | >1000 |
| CDK7/cyclin H/MAT1 | 67/19 | >100 |
| CDK12/cyclin K | 94/86 | >1000 |
| CDK13/cyclin K | 93/88 | >1000 |
| CDK14/cyclin Y | 70/39 | >100 |
| CDK16/cyclin Y | 23/5 | <100 |
| CDK17/cyclin Y | 30/7 | <100 |
| CDK18/cyclin Y | 53/12 | >100 |

**Supplementary Table 2: Results of GPCR agonist action testing of GFH009 administered at a maximum dose of 12 µM**

| Agonist action against GPCR type shown below | |
|---|---|
| **Target** | **% Agonist Effect @ Max Dose (12 µM)** |
| 5-HT1A (serotonin) | −0.33 |
| 5-HT1B (serotonin) | −51.01 |
| 5-HT2A (serotonin) | −1.68 |
| 5-HT2B (serotonin) | 4.55 |
| a1a (adrenoceptor) | 4.58 |
| a2a (adrenoceptor) | −3.87 |
| AD2 (adenosine) | 21.7 |
| b1 (adrenoceptor) | 4.53 |
| b2 (adrenoceptor) | −0.6 |
| CB1 (cannabinoid) | −0.7 |
| CB2 (cannabinoid) | 0.23 |
| CCKa | −0.4 |
| D1 (dopamine) | −1.49 |
| D2 (dopamine) | 3.66 |
| ETA (endothelin) | −0.08 |
| H1 (histamine) | 1.55 |
| H2 (histamine) | −0.03 |
| M1 (muscarinic Ach) | −2.18 |
| M2 (muscarinic Ach) | −0.63 |
| M3 (muscarinic Ach) | 0.49 |
| op-d (opioid) | −0.98 |
| op-k (opioid) | 21.57 |
| op-m (opioid) | 0.71 |
| V1A (vasopressin) | 0.59 |

**Supplementary Table 3: Results of off-target inhibitory action testing of GFH009 administered at a maximum dose of 10 µM**

| Competitive antagonist activity against GPCR type shown below | |
|---|---|
| **Target** | **% Inhibitory Effect @ Max Dose (10 µM)** |
| 5-HT1A (serotonin) | 12.31 |
| 5-HT1B (serotonin) | 4.71 |
| 5-HT2A (serotonin) | −7.10 |
| 5-HT2B (serotonin) | −24.98 |
| a1a (adrenoceptor) | 68.39 |
| a2a (adrenoceptor) | 5.16 |
| AD2 (adenosine) | −13.58 |
| b1 (adrenoceptor) | 30.48 |
| b2 (adrenoceptor) | −3.46 |
| CB1 (cannabinoid) | −9.21 |
| CB2 (cannabinoid) | −8.86 |
| CCKa | 16.26 |
| D1 (dopamine) | 1.10 |
| D2 (dopamine) | 10.34 |
| ETA (endothelin) | −0.32 |
| H1 (histamine) | 12.37 |
| H2 (histamine) | −18.49 |
| M1 (muscarinic Ach) | 98.78 |
| M2 (muscarinic Ach) | 95.69 |
| M3 (muscarinic Ach) | 99.12 |
| op-d (opioid) | −15.46 |
| op-k (opioid) | 11.54 |
| op-m (opioid) | 20.94 |
| V1A (vasopressin) | −1.57 |
